# Renal Cell Carcinoma Metastasis to the Penis: A Case Report and Literature Review

**DOI:** 10.3390/medicina60040554

**Published:** 2024-03-29

**Authors:** Dae Yeon Cho, Hyun Jung Kim, Jae Yoon Kim

**Affiliations:** 1 Department of Urology, Sanggye Paik Hospital, Inje University College of Medicine, 1342 Dongil-ro, Nowon-gu, Seoul 01757, Republic of Korea; waytogo@naver.com; 2 Department of Pathology, Sanggye Paik Hospital, Inje University College of Medicine, 1342 Dongil-ro, Nowon-gu, Seoul 01757, Republic of Korea; hjkim@paik.ac.kr

**Keywords:** renal cell carcinoma, metastasis, penis, kidneys, cancer, case report

## Abstract

Metastasis to the penis from renal cell carcinoma (RCC) or any other primary cancer site is unusual; when it does occur, it often involves multiple organs. A 75-year-old man presented with penile pain and swelling. Three months earlier, he had open radical nephrectomy with thrombectomy and was diagnosed with clear-cell RCC with tumor thrombosis in the inferior vena cava. The follow-up imaging indicated metastasis to the penis, prompting a total penectomy due to worsening pain. The excised mass displayed features consistent with metastatic RCC. This case underscores the need to consider rare metastatic sites, such as the metastasis of RCC to the penis, in RCC patients.

## 1. Introduction

About 85% of kidney tumors are classified as renal cell carcinoma (RCC), with approximately 70% showing clear cell histology [1]. RCC is a type of kidney cancer that primarily originates in the cells lining the small tubes (tubules) of the kidney.

In 2020, renal cell carcinoma (RCC) represented roughly 4.1% of all newly diagnosed cancer cases, with approximately 431,000 individuals diagnosed and 179,000 deaths worldwide, exhibiting the highest prevalence in Western countries [2]. A recent database revealed an annual average rise in RCC incidence of 0.6% [3].

Approximately 25–30% of patients will manifest metastatic disease at the point of initial diagnosis [4]. RCC can potentially metastasize, meaning it can spread to other parts of the body. The most common sites of RCC metastasis are the lungs, bones, liver, and brain. While it is theoretically possible for RCC to metastasize to the penis, it is an extremely rare occurrence [1,4]. Metastasis to the penis from RCC or any other primary cancer site is unusual, and when it does happen, it often involves multiple organs. In this report, we present an unusual case involving the metastasis of renal clear cell carcinoma to the corpora cavernosum of the penis.

## 2. Case Presentation

A 75-year-old patient presented with a complaint of pain and swelling in the penile region. The onset was gradual over the preceding month and he reported no history of trauma or significant changes in urinary habits.

**Figure 1:**
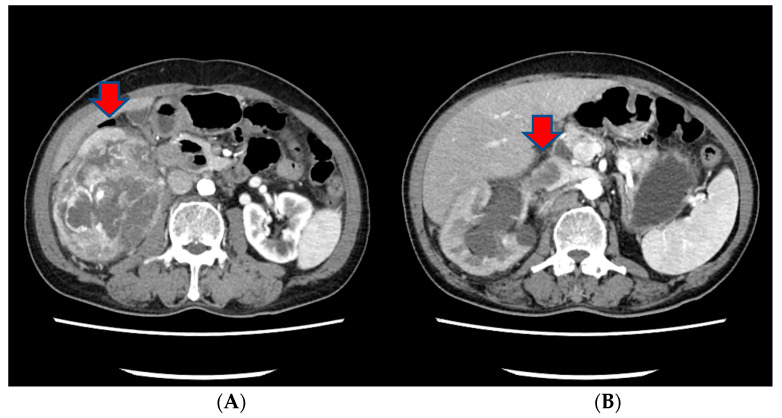
The CT scan of the abdomen of our patient. (**A**) An approximately 13 cm sized heterogeneous mass in the lower pole of the right kidney, exhibiting robust arterial enhancement. (**B**) The finding of tumor thrombosis in the inferior vena cava (IVC).

Approximately three months prior to presentation, he sought medical attention for right flank pain and underwent a computed tomography (CT) scan. Before the visit, the patient did not have any significant physiological or medical history. The findings revealed an approximately 13 cm sized heterogeneous mass in the lower pole of the right kidney, exhibiting robust arterial enhancement. It displayed invasion into the adjacent psoas muscle, indicating a potential RCC diagnosis. Additionally, evidence of tumor thrombosis in the inferior vena cava (IVC) was also observed (Figure 1). During the same CT examination, a specific finding of right scrotal varicocele was noted. This was likely attributed to thrombosis and extrinsic compression of the right gonadal vein due to the large mass in the right kidney. CT imaging of the chest showed no evidence of pulmonary mass or adenopathy.

The patient’s ECOG performance status score was 0, indicating full activity without restrictions. According to the American Joint Committee on Cancer (AJCC) TNM system, the patient’s stage grouping was T3N0M0, corresponding to stage III. The Mayo classification of the primary tumor thrombus in this patient was categorized as level I.

**Figure 2:**
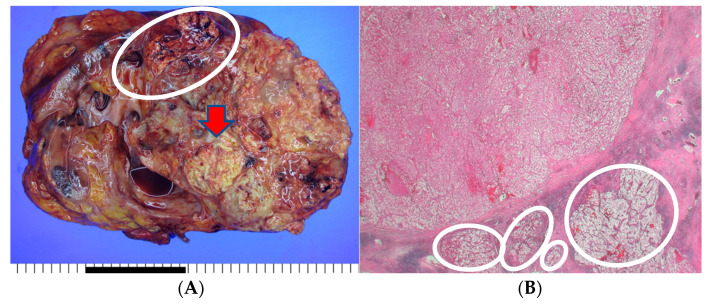

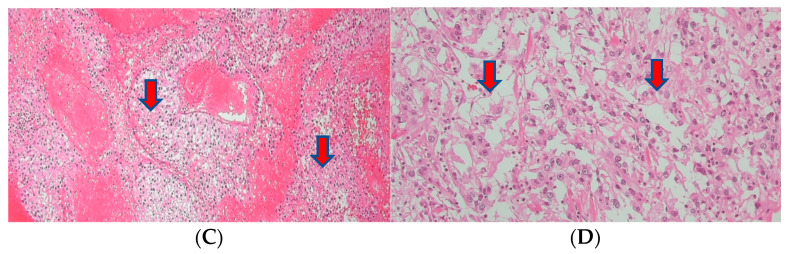
The histopathological findings of the resected kidney tissue and of tumor thrombosis in the inferior vena cava. (**A**) A renal mass (12.5 cm at its greatest dimension) replaces nearly the entire right kidney. The cut surface is diffusely necrotic (red arrow) with frequent tumor thrombi (white circle) at large vessels (the provided scale unit is 5 mm). (**B**) The tumor is partly encapsulated with multiple venous tumor invasions (white circles) (HE, ×10). (**C**) Separately sent tissue designated ‘inferior vena cava thrombi’ was involved with tumor cells (T3) (HE, ×40). (**D**) The tumor cells disclose clear/oncocytic cytoplasm (clear cell renal cell carcinoma) with Fuhrman nuclear grade 3/4. (HE, ×100).

The patient underwent an open radical nephrectomy with thrombectomy and was subsequently diagnosed with clear cell RCC with tumor thrombosis in the IVC upon microscopic and immunohistochemical examination (Figure 2).

**Figure 3:**
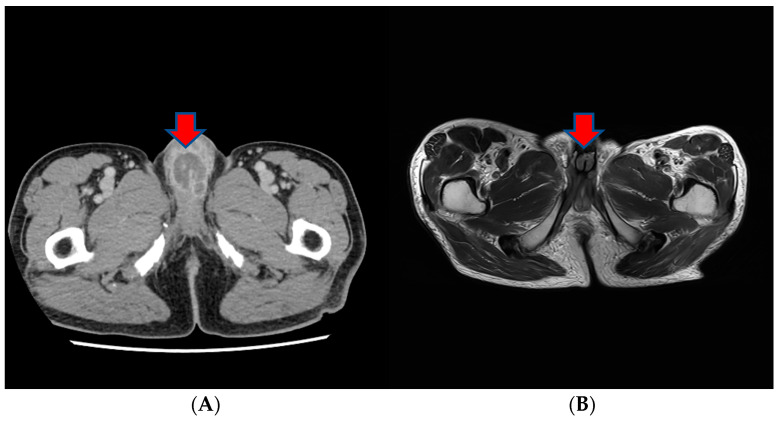

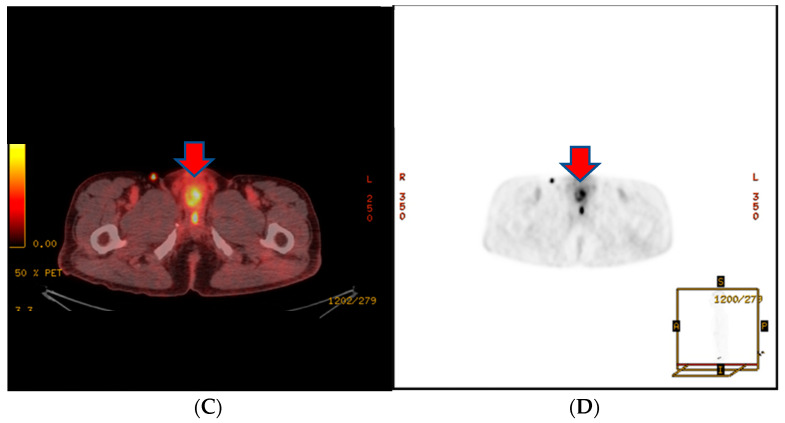
(**A**) An irregularly shaped enhancing mass in the penis on CT scan. (**B**) Magnetic resonance imaging (MRI) findings showing a heterogeneously enhanced mass-like lesion in the corpus cavernosa of the penis on T2-weighted images. (**C**,**D**) PET-CT showing an irregular increased fludeoxyglucose (FDG) uptake in the penis.

About a month after the surgical procedure, the patient began experiencing penile discomfort, which worsened over three months, prompting his hospital visit. Upon physical examination, a palpable mass was detected at the penoscrotal junction of the penis. The mass was tender, firm in consistency without ulcerations or rash, and measured approximately 5 cm in diameter. No other abnormalities were noted during the examination of the genitalia. Subsequent to this, a CT scan revealed an irregularly shaped enhancing mass in the penis, suggesting the possibility of metastasis (Figure 3). Laboratory results were within normal ranges. On urinalysis, pyuria was observed, but the urine culture showed no microorganism. Magnetic resonance imaging (MRI) findings revealed a lobulated contour and heterogeneously enhanced mass-like lesion in the corpus cavernosa of the penis on T2-weighted images, which raised the possibility of metastasis (Figure 3). A PET-CT showed an irregular increased fludeoxyglucose (FDG) uptake in the penis (Figure 3).

Following these results, a penile mass excision was performed for histological examination and removal of the tumor. Large yellow necrotic friable tissue was observed in the corpus cavernosa of the penis, and despite maximal removal efforts, the severe adhesions in the surrounding area and the depth of the tumor made complete excision impossible. The histopathological examination revealed metastatic clear cell carcinoma consistent with the patient’s previously diagnosed renal cell carcinoma.

In accordance with the Memorial Sloan Kettering (MSK) risk classification for metastatic RCC, the patient fell into an intermediate risk group, with one risk factor due to the time from initial diagnosis to systemic therapy initiation being less than one year.

After the penile mass excision, targeted therapy and radiotherapy were used to manage the remaining tumor. Sunitinib, a protein kinase inhibitor categorized as a targeted cancer medication, was administered once daily for about 4 weeks, spanning three cycles.

**Figure 4:**
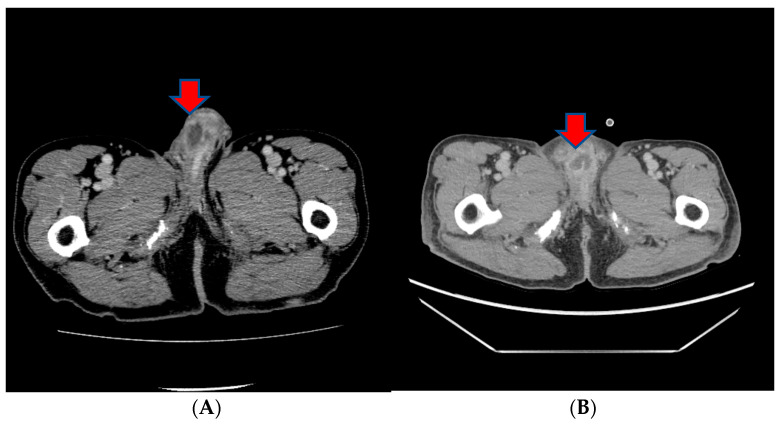
(**A**) A subsequent CT scan performed approximately 4 months later showing an increased size of the mass in the penis. (**B**) After radiation therapy, a follow-up CT scan showed the decreased size of the tumor.

However, in a subsequent CT scan performed approximately 4 months later, an increased size of the mass in the penis was observed, and persistent penile pain led to the consideration of radiation therapy (Figure 4). After radiation therapy, a follow-up CT scan showed a decreased tumor size (5.1 cm to 3.4 cm) (Figure 4).

However, owing to the patient’s ongoing and worsening pain, the decision was made to proceed with a total penectomy, followed by a perineal urethrostomy and the placement of a suprapubic cystostomy. Prior to performing a total penectomy, we conducted a cystoscopy. During this procedure, we identified signs of urethral stricture, likely attributed to infiltration from the corpus cavernosum and surrounding structures. The suprapubic cystostomy catheter was removed after one month, allowing the patient to urinate through the perineal urethrostomy.

**Figure 5:**
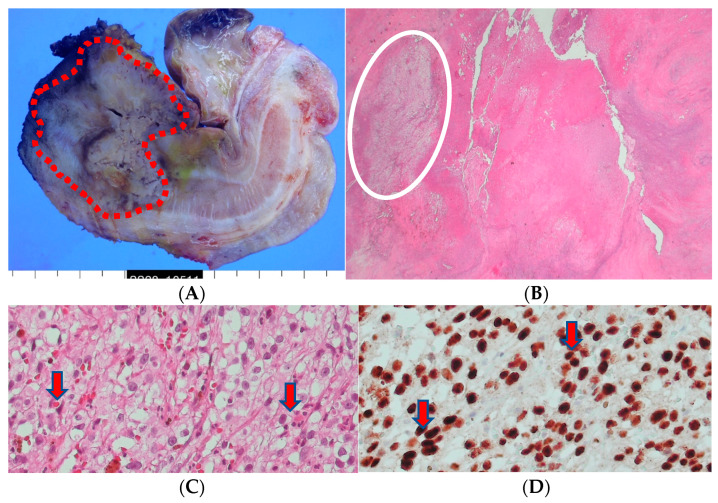
(**A**) A penectomy specimen showing an ill-defined nodular mass (4.3 × 3.2 × 2.5 cm) (round red circle) with extensive hemorrhage and necrosis (the provided scale unit is 5 mm). (**B**) The light microscopic finding disclosed a solid proliferation of clear cells with extensive necrosis (HE, ×10). (**C**) The composed tumor cells with clear cytoplasm (HE, ×200) and (**D**) with PAX-8 nuclear immunohistochemical expression (PAX-8, ×200).

The excised tissue displayed the presence of an ill-defined firm mass (4.3 × 3.2 × 2.5 cm) at the penile shaft. However, the urethra and other parts of the prepuce skin were largely unremarkable. Upon inspection of the cut surface of the mass, gray-yellowish and necrotic features were observed, infiltrating into the corpus cavernosum and surrounding structures (Figure 5). The histopathological examination confirmed the presence of metastatic renal cell carcinoma, clear cell type, with extensive necrosis and hemorrhage and a clear surgical resection margin (Figure 5).

Following a total penectomy, the patient did not experience significant discomfort and recovered without any notable complications. Follow-up imaging and laboratory tests will be conducted regularly to monitor for any recurrence or additional metastases.

## 3. Discussion

The occurrence of metastatic tumors in the penis is infrequent. Clinical manifestations encompass indurated nodules, mass formation, priapism, and ulceration [5]. As a general observation, secondary cancers affecting the penis are exceedingly rare, with approximately 300 reported cases documented over the past century. The prostate and bladder often serve as primary tumor sites, whereas the kidney constitutes the primary origin in only about 10% of all instances of secondary penile cancers [5,6].

The identification of RCC metastasis to the penis in this case raises several noteworthy considerations, both in terms of clinical implications and broader oncological insights. The metastasis of RCC to the penis is a rare occurrence, and its presentation as an isolated lesion is even more exceptional, with only isolated cases reported in the existing medical literature. The atypical presentation in this instance, where the metastasis was isolated to the penis, underscores the unpredictable nature of cancer’s spread.

The mechanisms underlying metastasis to distant and uncommon sites remain incompletely understood. The literature on RCC typically highlights the propensity for metastasis to organs such as the lungs, bones, liver, and brain.

This particular presentation lends credence to the hypothesis that hematogenous dissemination occurs through the invasion of the arterial system. Conversely, it is plausible that, owing to heightened intraabdominal pressure resulting from the substantial tumor size, tumor emboli selectively disseminate in a retrograde manner, from the renal vein to the pudendal veins and ultimately to the dorsal vein of the penis [7,8].

Among the documented cases, the left kidney was identified as the primary site of carcinoma in more than half of the instances [9]. However, in this patient, although the right kidney was identified as the primary site of carcinoma, the aggressive nature of the tumor and the presence of tumor thrombosis may have contributed to the occurrence of metastasis to the penis. In this case, there was also an observation of a finding of right scrotal varicocele, likely attributed to thrombosis and the extrinsic compression of the right gonadal vein due to the large mass in the right kidney. This could signify high pressure in venous flow and may have also acted as a contributing factor to the metastasis to the penis.

Several previous reports have suggested that, similar to this case, metastatic lesions of the penis secondary to malignant tumors typically involve the corpus cavernosa, and the invasion of the corpora spongiosum is rare [5]. However, Nezu et al. reported a case in which malignant priapism, as the initial clinical manifestation of metastatic RCC, involved both the corpora cavernosum and spongiosum [8].

Our patient presented with severe penile pain and dysuria, but without priapism. It is noteworthy that priapism, without pain, has been reported as the most frequent symptom of secondary cancer of the penis [5]. Priapism is most likely attributed to neoplastic invasion of the corpora and/or venous drainage system, which hinders the drainage of venous blood [10]. We hypothesize that the large penile tumor was causing a mass effect at the base of the penis, which irritated the dorsal nerve of the penis, leading to discomfort.

Localized RCC accounts for approximately 70% of new RCC diagnoses, with regional and distant metastasis comprising around 15% each [1]. Patients with resectable locoregional RCC often undergo surgical resection with curative intent, but up to 50% eventually progress to metastatic disease [11]. While nephrectomy remains the primary standard treatment for complete tumor removal, the role of adjuvant therapy post-surgery remains debatable. Tumor stage, grade, and regional nodal metastasis are critical prognostic factors, with patients exhibiting these factors considered at high risk for relapse and metastasis [12].

Extensive efforts have been dedicated to extending the clinical benefits of tyrosine kinase inhibitors (TKIs) and immunotherapy (IO) from the metastatic to the adjuvant setting, driven by the notably decreased survival rates in patients with relapsed or metastatic RCC [12]. While recent trials have shown successful outcomes with TKIs, there is still no firmly established consensus on adjuvant chemotherapy for RCC. Previous trials of TKIs in this setting have been unsuccessful [13,14].

Patients with high-risk RCC and the potential for recurrences, like this one, may consider adjuvant treatments such as Pembrolizumab. However, in Korea, as of 2023, adjuvant chemotherapy for RCC is entirely non-reimbursable. Consequently, patients must bear the full cost of the medication themselves. These policies place limitations on physicians in selecting available adjuvant treatments for RCC patients. The patient declined to undergo treatment with adjuvant therapies like Pembrolizumab due to its expensive cost and lack of insurance coverage.

Acquiring tissue for pathological examination is crucial in diagnosing metastatic RCC. After confirming the diagnosis, the treatment approach is guided by principles governing the management of metastatic RCC, encompassing surgical treatment, radiation therapy, chemotherapy, and/or targeted therapy. The decision for surgical intervention, including a total penectomy, was made considering the limited extent of the metastasis.

The prevailing consensus in most studies posits that metastasis to the penis arising from RCC typically signifies a more advanced stage of the disease and generally correlates with an unfavorable prognosis [15]. Despite the implementation of aggressive surgical interventions in conjunction with radiation therapy, chemotherapy, and/or targeted therapy, the reported maximum survival duration reached only sixteen months, in stark contrast to the 5-year survival rate of 10% in cases of metastatic RCC [15,16].

However, unlike the reported cases, the patient in this case reported a relief of symptoms after total penectomy. Although further follow-up is necessary, there are currently no signs of recurrence or additional metastases. This suggests that surgical treatment may be a more appropriate therapeutic approach when there is metastasis of RCC to the penis and no involvement of other organs.

The present case emphasizes the importance of clinical awareness and vigilance for atypical presentations, especially in patients with a history of RCC. Increased suspicion and thorough diagnostic evaluation are essential for the timely identification and management of uncommon metastatic occurrences. Regular physical examinations, particularly in such patients, can aid in promptly detecting uncommon masses. Additionally, it emphasizes the necessity of a multidisciplinary approach for optimal management in treatment. Certainly, exploring the molecular and biological factors linked to atypical metastatic patterns could enhance our comprehension of the disease and guide the development of targeted therapies.

## 4. Conclusions

This case highlights the importance of considering rare metastatic sites, such as metastasis of RCC to the penis, in patients with a history of RCC. Timely diagnosis and a comprehensive treatment strategy are essential for optimal patient outcomes. It is imperative to advance research efforts into these infrequent metastatic events.

## Data Availability

No new data were created or analyzed in this study. Data sharing is not applicable to this article.
